# A new multigene HCIQ subfamily from the sea anemone *Heteractis crispa* encodes Kunitz-peptides exhibiting neuroprotective activity against 6-hydroxydopamine

**DOI:** 10.1038/s41598-020-61034-x

**Published:** 2020-03-06

**Authors:** Aleksandra Kvetkina, Elena Leychenko, Victoria Chausova, Elena Zelepuga, Nadezhda Chernysheva, Konstantin Guzev, Evgeny Pislyagin, Ekaterina Yurchenko, Ekaterina Menchinskaya, Dmitry Aminin, Leonid Kaluzhskiy, Alexis Ivanov, Steve Peigneur, Jan Tytgat, Emma Kozlovskaya, Marina Isaeva

**Affiliations:** 1 G.B. Elyakov Pacific Institute of Bioorganic Chemistry, Far Eastern Branch of the Russian Academy of Sciences, 159, Pr. 100 let Vladivostoku, Vladivostok, 690022 Russia; 2 Department of Biomedical Science and Environmental Biology, Kaohsiung Medical University, 100, Shih-Chuan 1st Road, Kaohsiung, 80708 Taiwan; 3 V.N. Orekhovich Institute of Biomedical Chemistry, 10, Pogodinskaya St., Moscow, 119121 Russia; 4 Toxicology and Pharmacology, University of Leuven (KU Leuven), Campus Gasthuisberg, O&N2, Herestraat 49, P.O. Box 922, Leuven, B-3000 Belgium

**Keywords:** Biochemistry, Peptides

## Abstract

The Kunitz/BPTI-type peptides are ubiquitous in numerous organisms including marine venomous animals. The peptides demonstrate various biological activities and therefore they are the subject of a number of investigations. We have discovered a new HCIQ subfamily belonging to recently described multigene HCGS family of *Heteractis crispa* Kunitz-peptides. The uniqueness of this subfamily is that the HCIQ precursors contain a propeptide terminating in Lys-Arg (endopeptidase cleavage site) the same as in the neuro- and cytotoxin ones. Moreover, the *HCIQ* genes contain two introns in contrast to *HCGS* genes with one intron. As a result of Sanger and amplicon deep sequencings, 24 HCIQ isoforms were revealed. The recombinant peptides for the most prevalent isoform (HCIQ2c1) and for the isoform with the rare substitution Gly17Glu (HCIQ4c7) were obtained. They can inhibit trypsin with *K*_*i*_ 5.2 × 10^−8^ M and *K*_*i*_ 1.9 × 10^−7^ M, respectively, and interact with some serine proteinases including inflammatory ones according to the SPR method. For the first time, Kunitz-peptides have shown to significantly increase neuroblastoma cell viability in an *in vitro* 6-OHDA-induced neurotoxicity model being a consequence of an effective decrease of ROS level in the cells.

## Introduction

Kunitz-type proteinase inhibitors are present in various living organisms and in viruses. They are widely distributed and well-characterized in animals including marine invertebrates, snakes, spiders, ticks, flies and mammals^1^. In spider, snake, scorpion, cone snail and sea anemone venoms Kunitz-peptides may exist in multiple isoforms possessing conserved BPTI-like fold but exhibit different biological activities^2–9^. This phenomenon is associated with gene duplication and their diversification throughout adaptive evolution leading to the formation of families of evolutionarily related but functionally distinct genes^10^. Among sea anemone transcriptomes, such multigene families have been discovered in *Anemonia viridis*^11^, *Stichodactyla haddoni*^12^, and *Heteractis crispa*^5^. HCGS multigene family of *H. crispa* has been found to be divided in four distinct subfamilies (GS, RG, GG, and GN) forming the combinatory library of Kunitz/BPTI peptides^5^. The group of HCGN peptides was presented by one sequence, different from other sequences that are characterized by a propeptide insertion containing the cleavage site Lys-Arg, and additional residues Ile-Gln at the N-terminus of a mature peptide. Its full-length homolog HMIQ3c1, containing a mature peptide with the same residues at N-terminus, was derived from the cDNA of the sea anemone *Heteractis magnifica*^13^. The most abundant HCGS and HCRG peptides are being actively studied nowadays^14–17^. The main targets of Kunitz-peptides are serine proteinases, such as trypsin and α-chymotrypsin. Both HCGS and HCRG peptides characterized have been shown to inhibit trypsin. Some of them possess different biological activities besides serine proteinases inhibition. HCGS1.19, HCGS1.20 and HCGS1.36 suppress the increase of the Ca^2+^ response under the influence of histamine *in vitro*^14,17^ and provide analgesic action *in vivo*^18^; APHC1^19^ and HCRG21^16^ inhibit TRPV1. RmInI and RmInII exhibit anti-inflammatory activity by weakening rat inflammatory reactions in response to histamine injection^20^ while HCRG1 and HCRG2 reduce secretion of tumor necrosis factor-α (TNF-α) and interleukin 6 (IL-6), as well as expression level of IL-1β precursor (proIL-1β) in LPS-activated macrophages^15^. Moreover, HMIQ3c1 decreases β-amyloid-induced neurotoxicity on murine neuroblastoma cells^13^. Thus, Kunitz-peptides can be seen as potential therapeutic compounds preventing some inflammatory processes.

It is known that inflammation in cells can be triggered by oxidative stress, which also plays a major role in a number of neurodegenerative disorders including Parkinson’s disease (PD)^21,22^. One of the most widely used models for investigation of neuroprotective activity of different compounds is 6-hydroxydopamine (6-OHDA)-induced cytotoxicity. 6-Hydroxydopamine is a neurotoxin which destroys catecholaminergic systems by oxidative stress induced by non-enzymatically auto-oxidation to hydrogen peroxide and p-quinone^23^. At present, mainly low molecular compounds have been shown to exhibit neuroprotective activity^24–27^. Some peptides and proteins have been found to protect dopamine neurons and mitigate their destruction^28–30^. Recently, Kunitz-like peptide PcKuz3 from the zoanthid *Palythoa caribaeorum* has been shown to exert a neuroprotective effect in a 6-OHDA-induced neurotoxicity model on zebrafish larvae^31^. However, the ability of the sea anemone Kunitz-peptides to protect neurons against 6-OHDA toxicity has not been studied yet. Here we have investigated a new HCIQ gene subfamily encoding Kunitz-type proteinase inhibitors of *H. crispa*. We show that the recombinant HCIQ peptides inhibit serine proteinases as well as possess protective effect in a 6-OHDA-induced cytotoxicity model.

## Results

### Sequence identification and analysis of *H. crispa* Kunitz IQ peptide diversity

**Figure 1:**
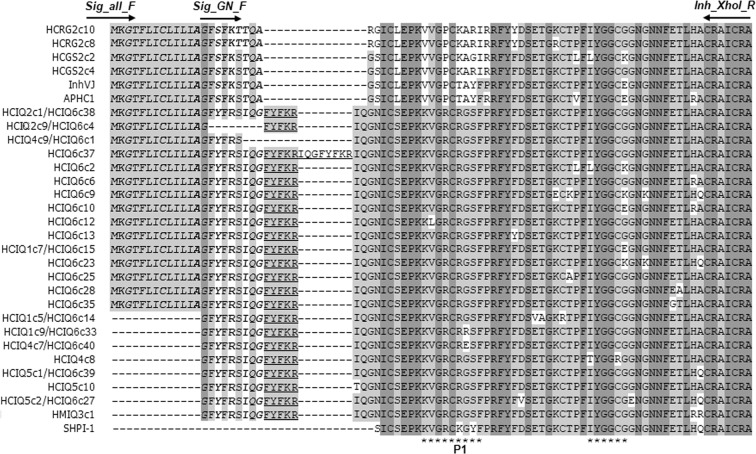
Multiple alignment of the precursor sequences of sea anemone Kunitz-type peptides. InhVJ^20,49^, APHC1^19,48^, HCIQ1c9-HCIQ6c38, HCRG2c8, HCRG2c10, HCGS2c2, HCGS2c4 from *H. crispa*; HMIQ3c1 from *H. magnifica*^13^; SHPI-1 from *Stichodactyla helianthus*^54^. The asterisks (*) below the sequences indicate the contact sites with serine proteinases. Р1 – amino acid residue of the inhibitor reactive center. Signal peptide is shown in *italic*. The pro-part is underlined. Identical and conservative residues are shown in light and dark grey respectively. Horizontal arrows indicate the primer directions.

To determine the full-length HCIQ coding sequences, we designed a pair of primers, SIG_all_F and Inh_XhoI_R, complementary to highly conserved regions encoding signal and mature parts of HCGS peptides, respectively. After PCR amplification, cloning, and sequencing we obtained cDNAs encoding HCGS precursors (HCGS2c2 and HCGS2c4), HCRG precursors (HCRG2c8 and HCRG2c10) and HCIQ precursors (HCIQ2c1 and HCIQ2c9) (Fig. 1). Deduced HCIQ2с1 of 85 aa comprises a signal peptide (22 aa), a propeptide (5 aa), and a mature peptide (58 aa). Deduced HCIQ2c9 differs from HCIQ2c1 by lacking a C-terminal part of the signal peptide. The first fifteen residues of the signal part are identical to those of HCGS and HCRG precursors, while the main differences are found in the remaining signal peptide (Fig. 1). The propeptide contains the proteolytic Lys-Arg cleavage site (at the positions 26–27). Thus, based on the high identity of an N-terminal part of the signal sequences of HCIQ, HCGS, and HCRG peptides, they belong to the same family. The differences in C-terminal signal regions and the presence of propeptide sequences allow us to single out the HCIQs as a distinct subfamily.

To evaluate the sequence diversity of HCIQ peptides, two approaches were applied. Firstly, we designed an additional primer (SIG_GN_F) complementary to the region encoding to the C-terminal part of the signal HCIQ peptide. As a result of PCR amplification and sequencing, we were able to obtain nine additional HCIQ isoforms (Fig. 1) which were similar to HMIQ3c1 (96–99% of identity). The most prevalent isoforms were HCIQ2c1 (68.75%) and HCIQ5c1 (12.5%); the rest of the sequences occurred in a single copy. The HCIQ4c9 precursor does not contain a propeptide similar to HCGSs, and has a mature peptide identical to HCIQ2c1. We suggest that *HCIQ4c9* is a transitional gene form between genes encoding HCGS and HCIQ peptides.

**Figure 2:**
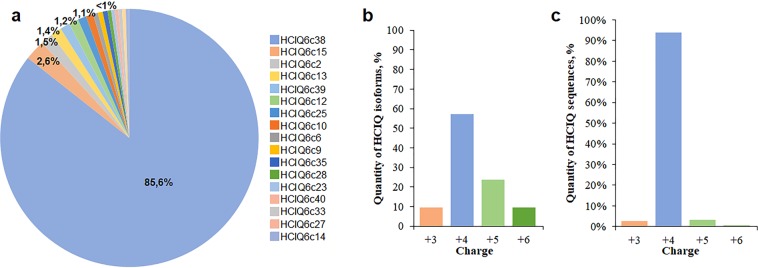
Diversity of isoforms derived from amplicon deep sequencing and charge distribution of HCIQ peptides. (**a**) Circular diagram of frequency of HCIQ isoforms derived from amplicon deep sequencing. The image was obtained by Microsoft Excel 14.0 program. (**b**) Distribution of HCIQ isoforms with different charge values. (**c**) Distribution of HCIQ-transcripts encoding the peptides with different charge values.

The second approach was to use next-generation amplicon deep sequencing. In total, 25201 reads were obtained. Among these 965 reads were HCIQ transcripts (3.83%); 764 reads with correct reading frame (79.1%), 174 reads with frameshift (18.1%), and 27 short reads (2.8%). All amplicon sequences encoded highly conserved signal and propeptides; only 68 reads contained synonymous substitutions in corresponding regions. The HCIQ6c37 amplicon contained repeats of a propeptide-coding region (Fig. 1). In total, 96 HCIQ mature peptide isoforms were deduced. The isoforms occurring less than in three copies (14.0%) were not taken into further analysis. The most abundant isoforms were HCIQ6c38 (520 amplicons), HCIQ6c15 (17 amplicons) and HCIQ6c39 (8 amplicons), the rest sequences included less seven copies (Fig. 2a). Comparison of the peptide sequences revealed that HCIQ peptides determined by Sanger sequencing except HCIQ4c8 and HCIQ5c10 were also identified by amplicon deep sequencing (Fig. 1).

All mature HCIQ peptides taken into analysis (22 isoforms, obtained by amplicon deep sequencing, nine of which were also derived by Sanger sequencing, and two isoforms obtained by Sanger sequencing only, Fig. 1) share high identity from 87 to 99% including six conservative Cys residues (except HCIQ1c5 and HCIQ4c8). The HCIQ peptides have two contact sites with serine proteinases: the main contact site (positions 11–19) and the weak contact site (positions 35–40). The HCIQ peptides contain Arg at the position P1 (named by HCIQ-R) unlike HCGS peptides with Arg (HCGS-R), Lys (HCGS-K), or Thr (HCGS-T) residues at this position (Fig. 1). The amino acid substitutions are concentrated near both contact sites but most of them are close to the weak contact site. The point substitutions Val12Leu in HCIQ6c12, Gly17Arg in HCIQ1c9 and Gly17Glu in HCIQ4c7 were found in the main contact site (Fig. 1). The substitutions such as Ile35 to Thr (HCIQ4c8) or Leu (HCIQ6c2), Cys39 to Arg (HCIQ4c8) as well as Gly40 to Glu (HCIQ6c10) or Lys (HCIQ6c2, HCIQ6c9 and HCIQ6c23) were at the weak contact site positions (Fig. 1). Compared to HCGS peptides, HCIQs contain unique substitutions such as Ile1, Gln2, Asn4, and Ser7.

HCIQ peptides have a narrow range of molecular masses (6258.09–6557.46 Da) and pI values (9.78–10.74) but a wide charge range (+ 2.82–+ 6.09) (Supplementary Table S1). The most prevalent HCIQ isoforms are those with the charge value + 4 (60%), the rest have the charge value + 3 (10%), + 5 (20%), and + 6 (10%) (Fig. 2b), while at the transcriptional level the number cDNAs encoding HCIQ peptides with charge value + 4 increases up to 95% (Fig. 2c).

### Determination of exon-intron gene structure

**Figure 3:**
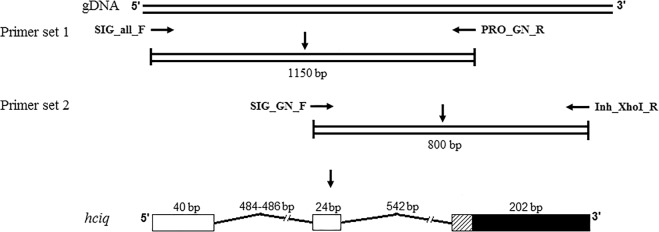
Scheme of HCIQ gene structure determination. The lengths of PCR fragments are shown below the lines. Horizontal arrows indicate the primer directions. The exons coding for signal peptide are displayed in white boxes; the exon coding for the pro-part and the mature peptide is shown in striped and black boxes respectively. The lengths of HCIQ gene elements are shown above the lines and boxes. The image was carried out using the Paint.net 4.0 program.

The exon-intron structure of *HCIQ* genes was determined by PCR amplifications of *H. crispa* genomic DNA (gDNA) with primer sets 1 (SIG_all_F and PRO_GN_R) and 2 (SIG_GN_F and Inh_XhoI_R) (Fig. 3). *H. crispa* cDNA was used as a template control. The lengths of PCR fragments obtained from gDNA were longer with an extra 1 kb for the primer set 1 and about 600 bp for the primer set 2 compared to those from cDNA. We compared the sequences and found that the HCIQ genes consisted of three exons. Intron 1 interrupts the conservative exons 1 and 2 encoding the signal peptide while intron 2 separates exon 3 coding the propart and mature peptide (Fig. 3). The intron/exon boundaries follow the GT/AG rule. The content of AT in the exons and introns oscillated slightly from 54.4% (exon 3) to 67.5% (exon 1). The intron identity was about 60%. The sequences of the first *HCIQ* and *HCGS* introns shared 93–96% identity.

### Phylogenetic analysis and electrostatic potential of HCIQ-peptides

**Figure 4:**
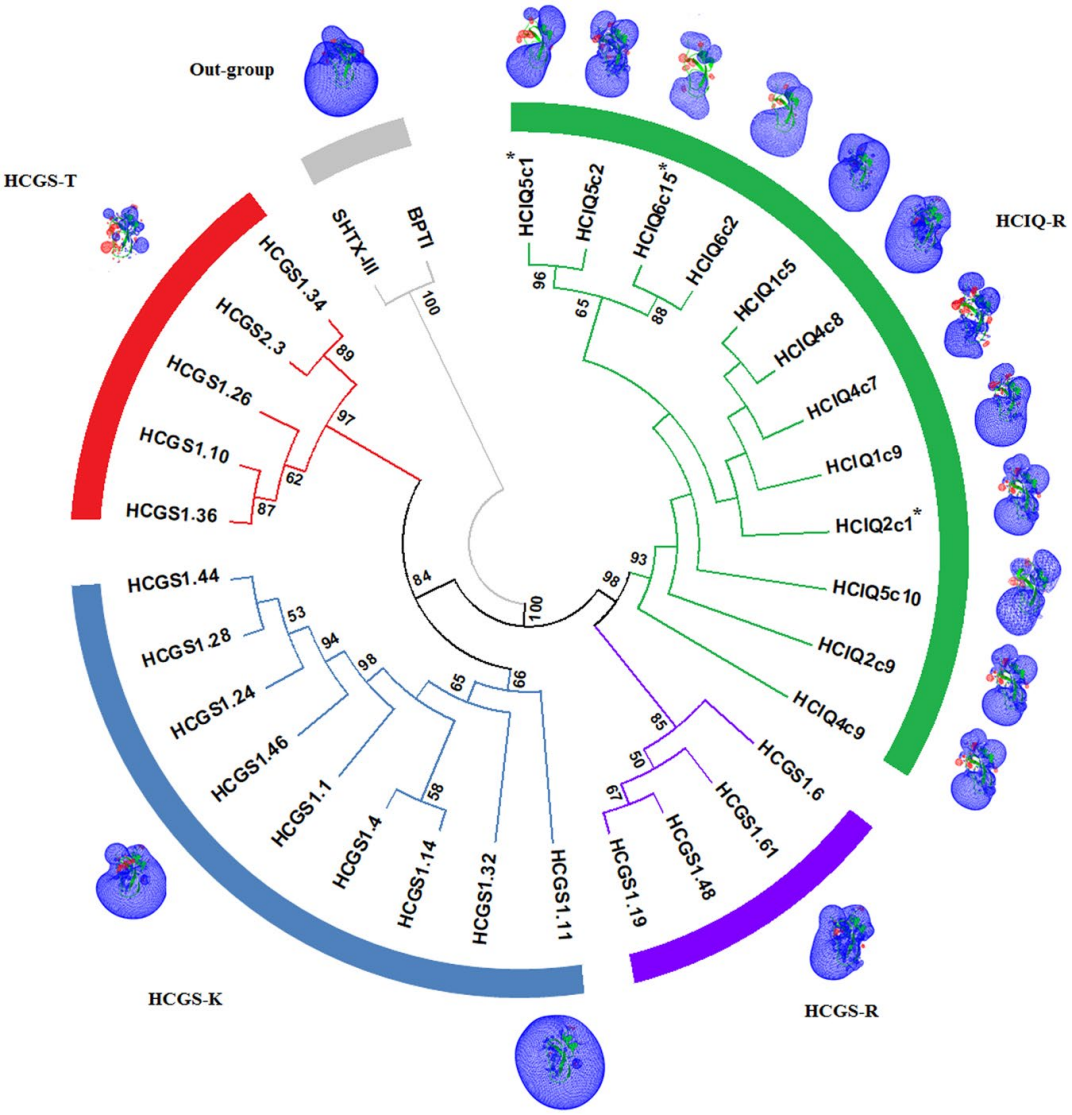
Phylogenetic analysis and electrostatic potential destribution of HCIQ and HCGS peptides. NJ phylogenetic tree was constructed by MEGA 6 using p-distance model and pairwise deletion with bootstrap support of 1000 replications. Nodes with confidence values greater than 50% are indicated. The abundant transcripts are shown with asterisks. Models of representative peptides are shown by green ribbon diagram, with an isopotential surface (blue color for positive charges and red for negative charges). The image was carried out using the SPDBV 4.1 (http://spdbv.vital-it.ch/) program^78^.

To explore the evolutionary relationships between HCIQ and HCGS precursors, we have analyzed their nucleotide sequences, containing more informative sites than the amino acid sequences. BPTI and SHTX-III were used as an out-group to construct a NJ phylogenetic tree. HCIQ and HCGS sequences share a common monophyletic origin (Fig. 4). The division into two groups is correlated with polymorphisms of the residue P1: the first group comprises HCIQ-R and HCGS-R clusters, while the second group includes HCGS-K and HCGS-T clusters. At the bottom of HCIQ-R cluster the precursor of HCIQ4c9 is located. Similarly to HCGS precursors, HCIQ4c9 has no propart region, which implies transition from HCGS to HCIQ through gene duplication. Therefore, in accordance with the monophyletic origin and the presence of the transitional gene form, we propose that the HCIQ peptides belong to the HCGS family. At the same time, we can assign the HCIQ-R cluster to a separate subfamily within the HCGS family, based on the high bootstrap values, the presence of the propart, and differences in the signal and mature sequences.

In order to determine how sequence divergence might influence functional specialization, we generated 3D-models of mature HCIQ peptides and calculated their electrostatic potential. The results of electrostatic potential clustering of HCIQ and HCGS peptides showed that both peptide groups were divided into the same four clusters as on the NJ phylogenetic tree, except HCIQ1c5 and HCIQ4c8, which fall into the BPTI cluster, and HCIQ1c9 that belongs to the HCGS-R cluster (Supplementary Fig. S1). Despite the high sequence and spatial structure similarity between HCIQ and HCGS peptides, they have diverse equipotential surfaces (Fig. 4). The majority of HCIQ peptides have a large positive electrostatic potential at the proteinase-binding loop. However, HCIQ4c7 has a local negative charge at the loop, due to Gly17Glu, which may imply a decrease in its proteinase inhibitory activity. In contrast, Gly17Arg in HCIQ1c9 results in an increase of the positive charge and falls into the HCGS-R cluster (Supplementary Fig. S1). Furthermore, the substitutions of Cys31Arg in HCIQ1c5 and Cys39Arg in HCIQ4c8 contribute to a high positive electrostatic potential intrinsic to BPTI cluster. Such electrostatic peculiarities of distinct HCIQ peptides may suggest the functional specialization.

### Expression and purification of recombinant HCIQ-peptides

To study the biological activity, we have chosen HCIQ2c1 and HCIQ4c7 with Gly17Glu at the proteinase-binding site. The recombinant HCIQ2c1 and HCIQ4c7 peptides were produced as fusion proteins with thioredoxin in *Escherichia coli* BL21 (DE3) cells. The fusion proteins with molecular masses about 23 kDa were isolated by metal-affinity chromatography and cleaved by CNBr. Recombinant HCIQ2c1 and HCIQ4c7 were purified by RP-HPLC. Retention times of the peptides on a reverse-phase column amounted to 34.9 min for rHCIQ2c1 and 27.3 min for rHCIQ4c7 (Supplementary Fig. S2). The final yields of rHCIQ2c1 and rHCIQ4c7 were 9.89 mg/l and 13.05 mg/l, respectively. The molecular masses were determined by MALDI-TOF/TOF and amounted to 6330 (rHCIQ2c1) and 6404 (rHCIQ4c7) Da, consistent with the predicted molecular masses. The N-terminal amino acid sequences (15 aa) determined by the automated Edman degradation matched well with amino acid sequences deduced from cDNA.

### Inhibitory activity of rHCIQ2c1 and rHCIQ4c7

The peptides rHCIQ2c1 and rHCIQ4c7 inhibit trypsin. The value of the inhibition constant (*K*_*i*_) of trypsin for rHCIQ2c1 (5.2 × 10^−8^ M) is closer to *K*_*i*_ values for the peptides in the groups with Arg and Lys at the position P1 (Table 1). At the same time, the value of *K*_*i*_ for rHCIQ4c7 (1.9 × 10^−7^ M) was the highest in the group with Arg but comparable with those of the peptides with Thr at the position P1.

**Table 1:** Trypsin *K*_*i*_ values for Kunitz-peptides from sea anemones.

Species	Peptide	Residue P1	Trypsin *K*_*i*_, M	Citation
*H. crispa*	rHCIQ4c7	R	1.90 × 10^−7^	
	rHCIQ2c1		5.20 × 10^−8^	
	rHMIQ3c1		5.00 × 10^−8^	^13^
	rHCGS1.19		3.00 × 10^−8^	^17^
*H. crispa*	APHC1	T	1.00 × 10^−6^	^19^
	rHCGS1.36		1.00 × 10^−7^	^17^
	rHCRG21		2.00 × 10^−7^	^16^
	rHCGS1.10		2.10 × 10^−7^	^17^
	APHC3		5.00 × 10^−7^	^48^
	APHC2		9.00 × 10^−7^	^48^
	InhVJ		7.38 × 10^−8^	^49^
*H. crispa*	HCRG1	K	2.80 × 10^−8^	^15^
	HCRG2		5.00 × 10^−8^	^15^
*S. helianthus*	SHPI-1		1.10 × 10^−10^	^47^

### Interaction of rHCIQ2c1 and rHCIQ4c7 with serine proteinases

The interaction of the peptides with serine proteinases (trypsin, α-chymotrypsin, human neutrophil elastase, pancreatic kallikrein and cathepsin G) was studied using the surface plasmon resonance (SPR) method. The peptides were immobilized on a CM4 sensor chip. It was determined that rHCIQ2c1 specifically binds to all target proteinases (Supplementary Fig. S3a), whereas rHCIQ4c7 bounds to trypsin and elastase only (Supplementary Fig. S3b). Kinetic parameters were calculated at 25 °C. Obtained data indicated the formation of tight complexes and eliminated non-specific binding (Table 2). The association constant (K_A_) magnitudes of the rHCIQ2c1/proteinase complexes were in the following order: trypsin >cathepsin G > α-chymotrypsin > elastase > kallikrein. The dissociation constant (*K*_D_) magnitudes of the complexes were in the range of 10^−10^–10^−7^ M; *K*_D_ of rHCIQ4c7 with trypsin being higher by three orders than for rHCIQ2c1, while binding with elastase differed insignificantly (Table 2).

**Table 2:** Kinetic parameters of complex formation between HCIQ peptides and proteinases.

Peptide	Proteinase	*k*_a_, M^−1^∙s	*k*_d_, s^−1^	*K*_A_, M^−1^	*K*_D_, M
rHCIQ2c1	Trypsin	2.05 × 10^4^	8.17 × 10^−3^	3.98 × 10^9^	2.51 × 10^−10^
	α-chymotrypsin	7.69 × 10^4^	7.49 × 10^−3^	1.03 × 10^7^	9.74 × 10^−8^
	Kallikrein	4.94 × 10^4^	3.79 × 10^−3^	2.04 × 10^6^	4.90 × 10^−7^
	Neutrophil elastase	6.80 × 10^4^	8.61 × 10^−3^	7.90 × 10^6^	1.27 × 10^−7^
	Cathepsin G	1.29 × 10^6^	3.59 × 10^−4^	3.58 × 10^9^	2.79 × 10^−10^
rHCIQ4c7	Trypsin	2.25 × 10^4^	4.66 × 10^−3^	4.83 × 10^6^	2.07 × 10^−7^
	Neutrophil elastase	1.19 × 10^4^	1.14 × 10^−2^	1.05 × 10^6^	9.55 × 10^−7^

Where *k*_a_ – association rate constants, *k*_d_ – dissociation rate constants, *K*_A_ – association constants, *K*_D_ – dissociation constants.

Thermodynamic parameters of intermolecular interactions were determined for the complexes of rHCIQ2c1 with trypsin and α-chymotrypsin in temperature range of 10–40 °С at an interval of 5 °С (Table 3). The magnitudes of free energy change (∆G) of the complexes differed slightly from the same complexes of HCRG1 and HCRG2 and were lower than for the InhVJ ones.

**Table 3:** Thermodynamic parameters of complexes of peptides with trypsin and α- chymotrypsin.

Peptide	Proteinase	∆H, kJ/mol	T∆S, kJ/mol	∆G, kJ/mol	Reference
HCIQ2c1	Trypsin	72.30	127.04	–54.70	
	α-chymotrypsin	19.58	63.27	–43.70	
HMIQ3c1	α-chymotrypsin	34.0	75.8	–41.8	^53^
HCRG1	Trypsin	33.00	88	–55	^15^
	α-chymotrypsin	28.00	77	–49	
HCRG2	Trypsin	39.00	92	–53	
	α-chymotrypsin	10.00	60	–50	
InhVJ	Trypsin	44.15	49.35	–5.2	^52^
	α-chymotrypsin	77.29	82.34	–5.5	

ΔG — Gibbs energy changes, TΔS — entropic term, and ΔH — enthalpy changes.

### Effect of rHCIQ2c1 and rHCIQ4c7 on K_v_ channels

**Figure 5:**
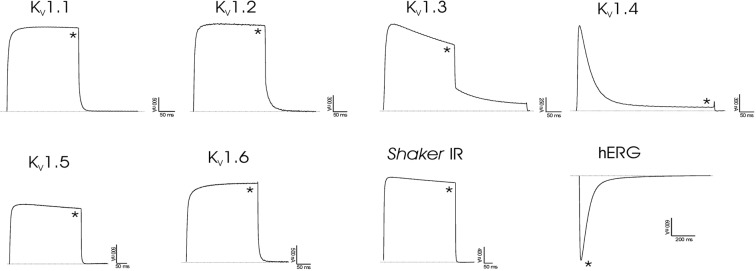
Differential effects of rHCIQ2c1 and rHCIQ4c7 on K_v_ isoforms expressed in *Xenopus laevis* oocytes. Representative whole cell K^+^ current traces of oocytes expressing cloned K_v_ isoforms K_v_1.1-K_v_1.6, hERG (K_v_11.1) and the *Drosophila* channel *Shaker* IR. The dotted line indicates zero-current level. The asterisk (*) indicates the steady-state current peak amplitude in the presence of 10 µM rHCIQ2c1 and rHCIQ4c7. The image was carried out by pClamp Clampfit 10.0 (Molecular Devices, Downingtown, PA, USA) and Origin 7.5 software (Originlab, Northampton, MA, USA).

The electrophysiological assays of rHCIQ2c1 and rHCIQ4c7 effects on eight cloned K_v_ channels (rK_v_1.1 – rK_v_1.6, *Shaker* IR, and hERG) were carried out. It was found that neither 10 μM of rHCIQ2c1 nor rHCIQ4c7 exerted any activity on the K_v_ channels (Fig. 5).

### Effect of rHCIQ2c1 and rHCIQ4c7 on 6-OHDA-induced cell death

**Figure 6:**
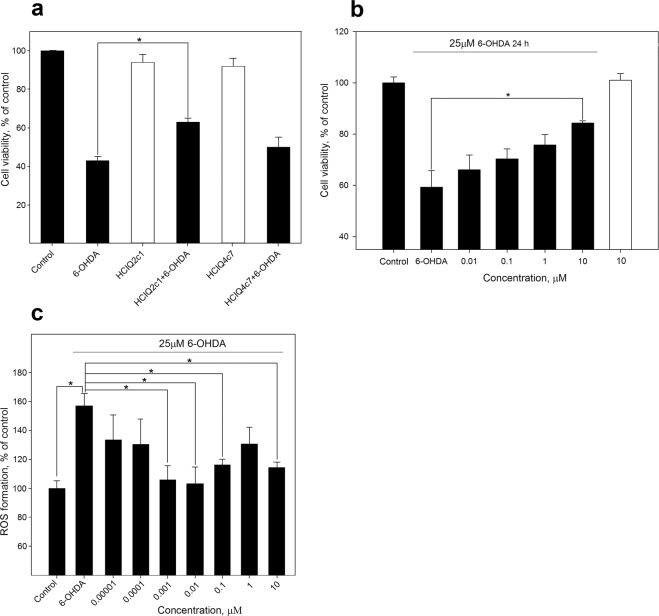
Effects of recombinant peptides on neuroblastoma cell viability with 6-OHDA. (**a**) The effect of 10 µM peptides on the viability of 6-OHDA-treated Neuro2a cells. (**b**) Dose-response effect of rHCIQ2c1 on Neuro2a cell viability. (**c**) Dose-response effect of rHCIQ2c1 on ROS formation into 6-OHDA-treated murine neuroblastoma cells. The influence of peptides on cell viability without 6-OHDA is shown in the white bar. * – p < 0.05.

Effects of rHCIQ2c1 and rHCIQ4c7 were studied using murine neuroblastoma Neuro2a cells in the presence of 6-OHDA (25 µM). After incubation of Neuro2a cells with recombinant peptides and 6-OHDA for 24 h, the cell viability was evaluated using MTT assay. As shown in Fig. 6a, rHCIQ4с7 and rHCIQ2с1 at concentrations of 10 μM were non-toxic and increased the cell viability by 14% and 47%, relative to cells treated with 6-OHDA respectively. The reliable effect was revealed for rHCIQ2c1. The cytoprotective effect of rHCIQ2с1 had a linear dose-dependent character and the maximum activity was achieved at concentration of 10 μM (Fig. 6b).

### Detection of intracellular ROS formation level

We estimated whether rHCIQ2c1 is able to inhibit reactive oxygen species (ROS) production. After incubation of Neuro2a cells with rHCIQ2c1 (up to 10 µM) and 6-OHDA (25 µM), ROS content was evaluated by measurement of dichlorofluorescin intensity. The highest ROS level was observed in the cells with 6-OHDA alone while the pretreatment with rHCIQ2c1 reduced ROS production by 34% to concentration of 0.001–0.01 µM. An increase in ROS formation when the concentration of rHCIQ2c1 increases from 0.01 to 1 µM, and its decrease again at 10 µM is probably due to complicated bell-shaped character of peptide influence upon intracellular ROS level (Fig. 6c).

## Discussion

The sea anemone *H. crispa* Kunitz-peptides form a combinatorial library, consisting of HCGS, HCRG, HCGG, and HCGN peptides coded by the *HCGS* multigene family^5^. The HCGN group was represented by only one sequence with propeptide insertion. The pro-part contained the subtilisin/kexin-like endoproteinase cleavage site (Lys-Arg) also found in precursors of actinoporins^32–34^ and neurotoxins^35–37^ involved in sea anemone venoms. Obviously, the mature HCIQ sequences start with Ile-Gln- (IQ-), rather than with Gly-Asn- (GN-) as suggested earlier, so, we designated the peptides as HCIQ where “HC” means *H. crispa* (Fig. 1). On the basis of PCR cloning and amplicon deep sequencing, we deduced 24 HCIQ peptides differing by both the precursor structure and point substitutions at the mature peptide. The most prevalent isoforms were HCIQ2c1, HCIQ6c15 and HCIQ5c1. The HCIQ precursors shared 88–98% identity to HMIQ3c1 derived from *H. magnifica* cDNA^13^. We revealed that HCIQs had four precursor structures differing by the presence of the propeptide (HCIQ2c1) or its tandem repeats (HCIQ6c37) and lacking the propeptide (HCIQ4c9) or C-terminal signal region (HCIQ2c9) (Fig. 1). Kunitz-type toxin genes of spider *Ornithoctonus huwena* also contain repeats in the precursor which, together with hypermutation and post-translational modifications, form the spider toxin diversity^38^. Importantly, a propeptide sequence has not been found in the known Kunitz-precursors of sea anemones while it has been described for their toxin precursors. The main function of a propeptide in the toxin precursors is the protection of its own tissues from crucial damages by these toxins. In conopeptides, the pro-part may be involved in folding and modification^39,40^. In addition, a short propeptide (6 aa) was also discovered in Kunitz-peptide precursors of *Conus californicus*^6^ and several spider species^4,41,42^ while the Lys-Arg cleavage site was found only in *C. californicus* precursors. We suggest that the appearance of the propeptide-coding region indicates that the HCIQ peptides could be involved in the sea anemone venom.

There is little information on gene organization of sea anemone Kunitz-homologs. Therefore, we determined the exon-intron structure of the *HCIQ* genes. We found that they included two introns in contrast to *HCGS* genes with one intron^5^. The first intron divided the sequences coding the signal peptide and the second intron separated the sequence coding the propeptide and the mature peptide (Fig. 3). Both *HCIQ* introns displayed low conservation between each other. Therefore, the first *HCIQ* intron shares high identity to *HCGS* intron including the presence of CA-repeat elements of different lengths. The CA-repeats can affect gene expression and participate in splicing regulation; long elements act as splicing enhancers while the short ones do as silencers^43,44^. The second *HCIQ* intron has no CA-repeats, and it is more variable than the first one. It strongly suggests that the second intron appeared because of the duplication of the first intron throughout the evolution of *HCIQ* genes.

According to the phylogenetic tree, *HCIQ* and *HCGS* genes originated from a common ancestor (Fig. 4). More precisely, *HCIQ* genes evolved following duplication of HCGS-R genes. This can be explained by the presence of a transitional gene form (HCIQ4c9), which is placed at the bottom of HCIQ-R cluster and does not code pro-part similarly to the *HCGS* genes. Therefore, we assert that *HCIQ* genes belong to the *HCGS* multigene family because of an absolute identity of N-terminal signal peptide encoding sequences. The presence of the second intron and pro-part encoding region in *HCIQ* genes as well as the variation of C-terminal signal and N-terminal mature regions of the precursors are likely to result from an adaptive evolution. This allows us to assign *HCIQ* genes to a separate subfamily within the *HCGS* multigene family.

To understand the functional specialization of HCIQ peptides, their electrostatic potential distribution was calculated. This characteristic is important to determine the specificity and kinetics of peptide molecular binding to the target^45^. Generally, the results of electrostatic potential clustering of HCIQ and HCGS peptides coincide with the phylogenetic data. Notably, two additional Ile-Gln residues on N-terminus do not contribute greatly to the charge distribution. The majority of HCIQ peptides have equipotential surfaces, similar to abundant HCIQ2c1 (Fig. 4). The peptide keeps a large positive charge at the proteinase-binding loop, typical for efficient Kunitz-type serine proteinase inhibitors^46,47^. Interestingly, HCIQ4c7 differed from HCIQ2c1 by a local negative charge in the loop (Gly17Glu) that may influence its biological activity.

Previous studies showed that Kunitz-peptides from *H. crispa* exhibit inhibitory activity against trypsin^13,14,17,20,48,49^. The HCIQ and HCGS peptides share high identity. The major differences are localized on N-terminus of the peptides (1, 2, 4, and 7 residues of HCIQs). The main and weak contact sites of HCIQ peptides have high residue conservation (Fig. 1). Both sites take part in the interaction with serine proteinases and define inhibitory specificity^50^. The main contact site is located on a relatively conserved binding loop and contains the residue P1, which makes a large part of enzyme-inhibitor contacts^47–51^. The residues of a weak contact site make additional electrostatic interactions and H-bonds with the proteinase active site^49^. The main contact site of HCIQ peptides completely coincides with the HCGS-R group whose representative, rHCGS1.19, inhibits trypsin (*K*_*i*_ 3.0 × 10^−8^ M)^17^. Furthermore, a high percentage of identity is observed between HCIQs and SHPI-1 from *S. helianthus* (87–94%), which inhibits serine, cysteine and aspartic proteinases along with blocking K_v_ channels^47^. Therefore, we suppose HCIQ peptides have to possess similar activities. At the same time, differences may indicate the direction to other targets.

For the investigation of biological activity, rHCIQ2c1 (the prevalent isoform) and rHCIQ4c7 (Gly17Glu) were obtained. Previous studies showed strong inhibitory activity of Kunitz-peptides was associated with a high positive charge at the proteinase-binding loop^51^. Therefore, the negatively charged amino acid residue at the main contact site of rHCIQ4c7 may decrease the ability to inhibit proteinases. Indeed, the inhibition constant value of trypsin for rHCIQ4c7 (*K*_*i*_ 1.9 × 10^−7^ M) is higher than those for rHCIQ2c1 (*K*_*i*_ 5.2 × 10^−8^ M) (Table 2). Despite high sequence identity of rHCIQ2c1 and rHCIQ4c7 with SHPI-1 (91–93%) from *S. helianthus*^47^, *K*_*i*_ for SHPI-1 is lower because there are differences in the main contact site residues including position P1.The study of the interaction of peptides with five serine proteinases by the SPR method showed that rHCIQ2c1 binds to all targeting proteinases (Supplementary Fig. S3a) in contrast to the characterized InhVJ, HCRG1 and HCRG2 from *H. crispa*^15,52^. rHCIQ4c7 is more specific than rHCIQ2c1; it binds to trypsin and human neutrophil elastase only (Supplementary Fig. S3b). The values of the entropic factor at both cases contributed to complex formation while the values of enthalpy indicated possible breakage of hydrogen and electrostatic bonds or changes of molecule conformation during the complex formation process^38^. Similar entropy-dependent complex formation was also observed for HMIQ3c1^53^, InhVJ^52^, HCRG1 and HCRG2^15^. According to the dissociation constant values, the most stable complexes are rHCIQ2c1/trypsin, rHCIQ2c1/cathepsin G and rHCIQ2c1/α-chymotrypsin. Both recombinant peptides interact with human neutrophil elastase with dissociation constant values around 10^−7^ M while other known Kunitz-peptides from *H. crispa* do not bind to the inflammatory proteinase^15,52^. Currently, the weak inhibitory activity against elastase has been shown also for SHPI-1^54^. Interestingly, rHCIQ2c1 binds to three inflammatory proteinases (cathepsin G, kallikrein and neutrophil elastase) indicating a potential anti-inflammatory activity.

We examined the effect of rHCIQ2c1 and rHCIQ4c7 in an *in vitro* model of 6-OHDA-induced cytotoxicity on murine Neuro2a cells. 6-OHDA is able to penetrate cells using dopamine (DAT) or norepinephrine (NET) transporters^55^ and stimulate ROS formation leading to an oxidative stress and cell death^56–58^. We revealed the peptides are capable to increase cell viability in the presence of 6-OHDA; the reliable effect is displayed for rHCIQ2c1 (Fig. 6a). This peptide decreased toxic activity of 6-OHDA in a dose-dependent manner (Fig. 6b) and effectively inhibited intracellular ROS formation (Fig. 6c). It was shown earlier that the Kunitz-like peptide, PcKuz3, from *P. caribaeorum* was able to suppress 6-OHDA-induced neurotoxicity in zebrafish larvae^31^. The authors hypothesized the effect is caused by blocking K_v_1 channels^31^. Indeed, K_v_ channels are actively involved in the regulation of neuronal processes and considered potential therapeutic targets for some neurodegenerative diseases, such as PD^59,60^. Thus, 4-aminopyridine, the blocker of K_v_1 and K_v_3 channel subfamily, suppresses MPTP-induced neurodegeneration and rat behavioral disturbances^61^; ShK-170 from *S. helianthus* is able to mitigate radiation-induced brain injury via K_v_1.3 blocking^62^. Moreover, Kunitz-type inhibitor SHPI-1 is also able to block K_v_1.1, K_v_1.2, and K_v_1.6^47^. However, neither rHCIQ2c1 nor rHCIQ4c7 exerted K_v_1 channel activity (Fig. 5). Consequently, the further investigation of the action mechanism of rHCIQ2c1 against 6-OHDA-induced neurotoxicity has to focus on the determination of the peptide interaction with dopamine transporter or enzymes involved in the synthesis and/or degradation of ROS.

In conclusion, here we have shown that paralogous genes coding Kunitz-peptides in *H. crispa* genome form distinct subfamilies within a great multigene family. The appearance of a propeptide in HCIQ precursors may be necessary for the employment of the peptides in sea anemone venom in enhancing toxic effects*.* It has been revealed for the first time that Kunitz-peptides are able to decrease the neurotoxic effect of 6-OHDA on neuroblastoma cells by reducing the ROS level. Taking into account their specificity to target proteinases, including inflammatory proteinases, we suppose that sea anemone Kunitz-peptides possess a great therapeutic potential in the complex treatment of neurodegenerative diseases.

## Materials and Methods

### Animal collection

*Heteractis crispa* specimens were collected near Vietnam during a marine expedition aboard the research vessel “Academik Oparin”. Dr. E. Kostina (National Scientific Center of Marine Biology of the Far Eastern Branch of the Russian Academy of Sciences, Vladivostok, Russia) confirmed the identity of the species.

### Precursor and gene structure determination

PCR amplification of sequences encoding HCIQ peptides was carried out using gene specific forward primers SIG_all_F (5′- CAA AGA CAA GAT AAC AAG ATG AAG GGA-3′) and SIG_GN_F (5′-AGG TTT CTA TTT CAG AAG CAT TCA AGG T-3′) complementary to 5′- and 3′-tereminal sequences coding signal peptide, respectively, and reverse primer Inh_XhoI_R (5′-ACT CGA GTT ACG CCC TGC ATA TAG CTC GGC AT-3′) complementary to 3′-terminal sequence coding mature peptide. PCR was conducted using GoTaq DNA Polymerase (Promega, USA) under the following conditions: 95 °C for 2 min; 30 cycles of 94 °C 30 s; 60 °C 30 s; 72 °C 1 min; 72 °C 3 min. Complementary DNA isolated earlier^63^ was used as a template. PCR-fragments (∼250 bp) were analyzed by gel electrophoresis, purified, cloned into the pTZ57R/T vector (Thermo Fisher Scientific, USA), and transformed into Top 10 *E. coli* cells (Invitrogen, Life Technologies) according to standard protocols^64^. Positive colonies were screened by amplifications with M13 universal primers. Plasmids containing inserts were sequenced using the ABI 3130xl Genetic Analyzer (Applied Biosystems, USA) according to the manufacturer protocol.

Genomic DNA (gDNA) was isolated from *H. crispa* tentacles by homogenization in guanidinium thiocyanate followed by phenol-chloroform extraction^64^ and used as a template to identify introns into coding region of HCIQ genes. PCR amplifications were conducted using two gene specific primer sets: first one, Inh_SIG_all_F and Inh_PRO_GN_R (5′-ATG TTA CCT TGA ATC CTT TTG-3′) complementary to sequences encoding propart; the second one, SIG_GN_F and Inh_XhoI_R. Complementary DNA was used as control. PCR amplification, cloning, and sequencing were carried out as described above.

### Amplicon deep sequencing

The sequence diversity of HCIQ-peptides of sea anemone *H. crispa* was investigated with amplicon deep sequencing using a ‘One-Way Reads’ experimental design according to Guidelines for Amplicon Experimental Design (Roche/454 Life Sciences). To generate amplicons, cDNA sample isolated from *H. crispa* was amplified by PCR with fusion primers A_Mid1sig_allF (5′-CGT ATC GCC TCC CTC GCG CCA **TCA G**AC ACG ACG ACT CAA AGA CAA GAT AAC AAG ATG AAG GGA-3′) and B_inh_R (5′-CTA TGC GCC TTG CCA GCC CGC **TCA G**CT AAC AAG ATT ACG CCC TGC ATA-3′), including a 21-mer adaptor sequences, sequencing key’TCAG‘, 11-base molecular barcode RL001 and template-specific primer complementary to signal part of HCGS-peptides, and GoTaq DNA Polymerase (Promega, USA) under the following conditions: 95 °C for 3 min; 35 cycles of 94 °C 30 s, 55 °C 30 s, 72 °C 1 min; 72 °C 15 min. Amplicons were analyzed by gel electrophoresis, purified and quantified using the Quant-iT PicoGreen dsDNA reagent (Invitrogen, Life Technologies). Emulsion PCR, bead recovery, bead enrichment and sequencing on a Genome Sequencer Junior (Roche/454 Life Sciences) were performed using the GS Junior Titanium series sequencing reagents and corresponding protocols. Data analysis was performed using the GS Amplicon Variant Analyzer (AVA) version 2.7 (Roche/454 Life Sciences) and HCIQ2с1 sequence was used as the alignment reference.

### Sequence, phylogenetic analysis and modeling

Determined nucleotide and amino acid sequences were aligned by MEGA 6^65^ and Vector NTI (Invitrogen, Life Technologies) programs. Searching of the Kunitz/BPTI homologs was carried out in the GeneBank database using BLASTN and BLASTP algorithms (http://www.ncbi.nlm.nih.gov/BLAST). The phylogenetic analysis of HCIQ precursor nucleotide sequences was conducted in MEGA 6^65^, using the neighbor-joining method^66^ with a bootstrap test^67^ of 1000 replicates. The evolutionary distances were computed using the p-distance method^68^. BPTI from *B. taurus* and SHTX-III from *S. haddoni* were used as an out-group. Alignment gaps were excluded using function “Pairwise deletion”. The spatial structure models of HCIQ peptides were generated using Modeller 9.11 and Chimera 1.9^69,70^ programs. The atomic coordinates of SHPI-1 (PDB ID 1SHP) from the sea anemone *S. helianthus* used as a template (the identity between SHPI-1 and HCIQs is 87–94%). The quality of the models was tested using a web server PROCHECK and MOE program (Chemical Computing Croup, Inc.; http://www.chemcomp.com/). The electrostatic potential distribution of Kunitz peptide models was computed and equipotential surfaces were visualized using program SPDBV 4.1 (http://spdbv.vital-it.ch/). Clustering of HCIQ and HCGS peptides based on electrostatic properties was conducted using webPIPSA server (http://pipsa.h-its.org/)^45^. Calculation of HCIQ peptide molecular masses was performed using the software Vector NTI 10 (Invitrogen, Life Technologies). Total net charges and pI values of these peptides were computed by PROPKA 2.0 program^71^ on the basis of 3D-structure models.

### Cloning, expression and purification of HCIQ-peptides

To amplify sequences encoding mature HCIQ peptides gene-specific forward primer GN_EcoR_F (5′-GCG AAT TCG ATG ATT CAA GGT AAC ATT TGT TCA-3′) containing EcoRI restriction site and Met-codon for CNBr cleavage and reverse primer Inh_XhoI_R (5′-ACT CGA GTT ACG CCC TGC ATA TAG CTC GGC AT-3′) containing XhoI restriction site and stop-codon were used. PCR of the DNA-fragments built in pTZ57R/T vector (Thermo Fisher Scientific, USA) was carried out using GoTaq DNA Polymerase (Promega, USA) under the following conditions: 95 °C for 3 min; 30 cycles of 94 °C 30 s, 55 °C 30 s, 72 °C 1 min; 72 °C 15 min. The recombinant plasmids pTZ57/*hciq2c1* and pTZ57*/hciq4c7* were used as templates. PCR-fragments (∼200 bp) were restricted by EcoRI and XhoI endonucleases and cloned into the pET32b(+) vector (Novagen, Germany). After verification by sequencing of the recombinant plasmids were transformed into BL21 (DE3) *E. coli* cells (Novagen, Germany) for expression.

Cells transformed with the recombinant plasmids were cultured at 37 °C in Luria-Bertani medium containing 100 µg/ml ampicillin up to reaching the optical density (OD600) ~0.5. After induction with IPTG in concentrations of 0.2 mM for rHCIQ2c1 and 0.3 mM for rHCIQ4c7, the cells were incubated at 37 °C for 3 hours at 180 rpm, centrifuged for 6 min at 6000 rpm at 4 °C, and supernatant was removed. Occurrence of recombinant peptides was determined in 12% polyacrylamide gel by Laemmli’s SDS-PAGE method^72^. Precipitate was resuspended in the start buffer for affinity chromatography (400 mM NaCl, 20 mM Tris-HCl buffer, pH 8.0) and ultrasonicated on ice. Then lysed cells were centrifuged for 10 min at 10000 rpm to remove all insoluble particles. Supernatant was applied to a Ni-NTA agarose (Qiagen, Netherlands) and fusion protein was purified under native conditions according to the manufacturer’s instructions. The collected fusion protein was cleaved by CNBr overnight at room temperature with molar ratio CNBr to protein 600:1. The recombinant peptides were purified from reaction mixture on reverse-phase column Jupiter C_4_ 10 × 250 mm (Phenomenex, USA) using a linear gradient of acetonitrile (from 0% to 70%) with 0.1% TFA in 70 min with a constant flow rate of 1.5 ml/min. The molecular masses of the purified peptides were analyzed by Ultra Flex III MALDI-TOF/TOF mass spectrometer (Bruker, Germany). The amino acid sequences of the recombinant peptides were determined on an automated sequencer protein Procise 492 Clc (Applied Biosystems, Foster City, CA, USA).

### Trypsin inhibitory activity

The trypsin inhibitory activity of HCIQ peptides was estimated according to the standard procedure using N-α-benzoyl-D,L-arginine p-nitroanilide (BAPNA) as a substrate^73^. The trypsin inhibition constants of rHCIQ2c1 and rHCIQ4c7 were determined by the method of Dixon^74^ using substrate (BAPNA) concentrations of 0.6 and 1.2 mM. The trypsin concentration in the reaction mixture was 208 nM. The range of peptide concentrations was 0–26.6 µM for rHCIQ2c1 and 0–24 µM for rHCIQ4c7. The constants (*K*_*i*_) were calculated based on the results of three parallel experiments. Computational error limits were in the range of 0.1–0.5%.

### SPR measurements

The study of the interaction of serine proteinases with rHCIQ2c1 and rHCIQ4c7 was performed on SPR biosensor Biacore 3000 (GE Healthcare, USA) running under the program “Biacore 3000 Control Software v.1.0”. The evaluation of data was carried out using “Biacore Evaluation v.4.1”. Peptides were covalent immobilized on carboxymethylated surface of the Biacore CM4 sensor chip (GE Healthcare, USA) activated by EDC/NHS (N-hydroxysuccinimide/1-ethyl-3-(3-dimethylaminopropyl)carbodiimide hydrochloride) mixture by the injection of peptide solution (100 µg/ml) in 10 mM sodium acetate (pH 5.5) during 10 min with flow rate 5 µl/min. Quantities of immobilized peptides were 970 RU (resonance units, 1 RU corresponds 1 pg peptide) for rHCIQ2c1 and 460 RU for rHCIQ4c7. In all of the experiments, the flow cell 1 (Fc1) without the immobilized polypeptides was considered as the reference cell for the correction of the signal responses. HBS (HEPES buffered saline-NaCl): 0.15 M NaCl, 0.01 M HEPES, pH 7.4 (cat. no. BR-1003-69, GE Healthcare, USA) was used as the running buffer for the SPR assays. Protease solutions at concentrations from 1 nM to 5 μM were passed through measuring and control biosensor cells at 25 °C during 5 min with a flow rate 5 µl/min. Further complex breakdown was recorded during at most 10 min. After each cycle of SPR measurement, the sensing surface was regenerated by the injection of 50 mM NaOH for 0.5 min at a flow rate of 50 μl/min. Changes in Gibbs free energy (ΔG) at 25 °C were calculated by the following equation:

1 $$\Delta G={\rm{RTln}}{K}_{D},$$

where *K*_D_ is the dissociation constant. Changes in enthalpy (ΔH) and entropic term (−TΔS) were calculated from the linear equation:

2 $$\Delta G=\Delta H-{\rm{T}}\Delta {\rm{S}},$$

using the liner approximation of temperature dependence of ΔG (Van’t Hoff diagram).

### Expression of voltage-gated ion channels in *X. laevis* oocytes

For the expression of the voltage-gated potassium channels (rK_v_1.1, rK_v_1.2, hK_v_1.3, rK_v_1.4, rK_v_1.5, rK_v_1.6, *Shaker* IR and hERG) in *X. laevis* oocytes, the linearized plasmids were transcribed using the T7 or SP6 mMESSAGE-mMACHINE transcription kit (Ambion, USA). The harvesting of stage V–VI oocytes from an anaesthetized female *X. laevis* frog was previously described^75^. Oocytes were injected with 50 nl of cRNA at a concentration of 1 ng/nl using a micro-injector (Drummond Scientific, USA). The oocytes were incubated in a solution containing (in mM): NaCl, 96; KCl, 2; CaCl2, 1.8; MgCl_2_, 2; HEPES, 5 (pH 7.4), supplemented with 50 mg/l gentamycin sulfate.

### Electrophysiological recordings

Two-electrode voltage-clamp recordings were performed at room temperature (18–22 °C) using a Geneclamp 500 amplifier (Molecular Devices, USA) controlled by a pClamp data acquisition system (Axon Instruments, USA), as described in^47^. Whole cell currents from oocytes were recorded 1–4 days after injection. Bath solution composition was ND96 (in mM): NaCl, 96; KCl, 2; CaCl_2_, 1.8; MgCl_2_, 2 and HEPES, 5 (pH 7.4). Voltage and current electrodes were filled with 3 M KCl. The resistances of both electrodes were kept between 0.7 and 1.5 MΩ. The elicited currents were filtered at 0.5 kHz and sampled at 2 kHz using a four-pole low-pass Bessel filter. Leak subtraction was performed using a -P/4 protocol. K_v_1.1–K_v_1.6 and *Shaker* currents were evoked by 250 ms depolarizations to 0 mV followed by a 250 ms pulse to −50 mV, from a holding potential of −90 mV. Current traces of hERG channels were elicited by applying a + 40 mV prepulse for 2 s followed by a step to −120 mV for 2 s. Comparison of two sample means was made using a paired Student’s test (p < 0.05). All data represent at least three independent experiments (n ≥ 3) and are presented as mean ± standard error. All data were analysed using pClamp Clampfit 10.0 (Molecular Devices, Downingtown, PA, USA) and Origin 7.5 software (Originlab, Northampton, MA, USA).

### 6-Hydroxydopamine-induced cytotoxicity model

The cytoprotective activities of peptides was examined, as described previously^76^. Neuro2a murine neuroblastoma cells (ATCC CCL-131) (American Type Culture Collection, Virginia, USA) were cultivated in DMEM medium (BioloT, Russia) with 10% of fetal bovine serum (FBS, BioloT, Russia) and 80 µg/ml gentamicine (BioloT, Russia). The cells were seeded onto 96-well plate at concentration of 1 × 10^3^ cells per well. Substances were added at different concentrations, and then cells were incubated for 30 min  at 37 °C and 5% CO_2_. Cells were treated with 6-OHDA (25 μM) for 24 h at 37 °C with 5% CO_2_. Thereafter, medium was replaced with clean serum-free medium, and cells were treated with 3-(4,5-dimethylthiazol-2-yl)-2,5-diphenyltetrazolium bromide (MTT) for 4 h^77^. Then the medium was collected and whole cells were lysed in 100% dimethylsulfoxide (DMSO), the absorbance was measured at 570 nm using Multiscan FC (Thermo Fisher Scientific, USA). Cytotoxic activity of substance was calculated as concentration of 50% metabolic cell activity inhibition (IC_50_) . The results were expressed as the mean ± SD of three independent experiments.

### Analysis of ROS production

The intracellular ROS level was detected using 2,7-dichlordihydrofluorescein diacetate (H2DCF-DA) solution (Molecular Probes, final concentration 10 µM). In this study, mouse neuroblastoma Neuro2a cells seeded on 96-well plates were washed twice with serum-free DMEM and thereafter incubated for 1 h in serum-free MEM medium in the presence of compounds in concentrations from 10 pM to 10 µM. Cell were treated with 6-OHDA (25 μM) for 30 min at 37 °C with 5% CO_2_. Followed by treatment with 6-OHDA, the cells were washed once with PBS. After washing with serum-free DMEM medium, the cells were loaded with H2DCF-DA for 30 min. The intensity of dichlorofluorescin fluorescence was measured with plate reader PHERAstar FS (BMG Labtech, Germany) at λex = 485 nm and λem = 518 nm. The cytoprotective effect of peptides and the increase in cell viability under their action was calculated as a percentage, taking the cell viability treated with 6-OHDA as 100%.

### Accession codes

sequences obtained by Sanger sequencing were submitted to GeneBank database under accession numbers MH249934–MH249943.

## Supplementary information

Supplementary Information.

## Data Availability

The additional data that support the manuscript are available from the corresponding author upon request.
