# Supplementary material for: A new multigene HCIQ subfamily from the sea anemone Heteractis crispa encodes Kunitz-peptides exhibiting neuroprotective activity against 6-hydroxydopamine
Source: Sci Rep. 2020 Mar 6;10:4205. doi: 10.1038/s41598-020-61034-x (PMC7060258; doi:10.1038/s41598-020-61034-x)
Supplement: Supplementary file 1 — Supplementary Information. [file 41598_2020_61034_MOESM1_ESM.pdf]

Supplementary Information

A new multigene HCIQ subfamily from the sea anemone *Heteractis crispa* encodes Kunitz-peptides exhibiting neuroprotective activity against 6-hydroxydopamine

Aleksandra Kvetkina[1], Elena Leychenko[1,*], Victoria Chausova[1], Elena Zelepuga[1], Nadezhda Chernysheva[1], Konstantin Guzev[1], Evgene Pislyagin[1], Ekaterina Yurchenko[1], Ekaterina Menchinskaya[1], Dmitry Aminin[1,2], Leonid Kaluzhskiy[3], Alexis Ivanov[3], Steve Peigneur[4], Jan Tytgat[4], Emma Kozlovskaya[1], and Marina Isaeva[1]

Peptide	Molecular mass, Da	Charge under pH 7	Number of charged residues		pI
			Positive	Negative	
HCIQ1c5	6324.19	+5.57	9	3	10.74
HCIQ1c9	6429.29	+4.66	9	4	10.18
HCIQ1c7	6402.22	+3.09	8	5	9.78
HCIQ2c1	6330.15	+3.84	8	4	10.73
HCIQ4c7	6404.10	+2.82	8	5	10.17
HCIQ4c8	6372.15	+4.73	9	4	10.19
HCIQ5c1	6387.21	+4.09	8	4	9.96
HCIQ5c2	6443.31	+3.87	8	4	10.55
HCIQ5c10	6318.10	+4.09	8	4	10.02
HCIQ6c2	6417.32	+5.09	9	4	10.08
HCIQ6c6	6349.2	+4.99	9	4	10.12
HCIQ6c8	6316.13	+4.09	9	4	9.96
HCIQ6c9	6557.46	+5.09	10	5	10.04
HCIQ6c10	6421.26	+ 4.00	9	5	9.96
HCIQ6c12	6344.18	+4.09	8	4	9.96
HCIQ6c13	6346.15	+4.09	8	4	9.89
HCIQ6c20	6332.13	+4.09	8	4	9.89
HCIQ6c23	6529.45	+6.09	10	4	10.18
HCIQ6c25	6300.13	+4.09	8	4	9.96
HCIQ6c28	6300.13	+4.09	8	4	9.96
HCIQ6c35	6258.09	+5.08	8	3	10.12

Table S1. Physicochemical characteristics of HCIQ peptides.

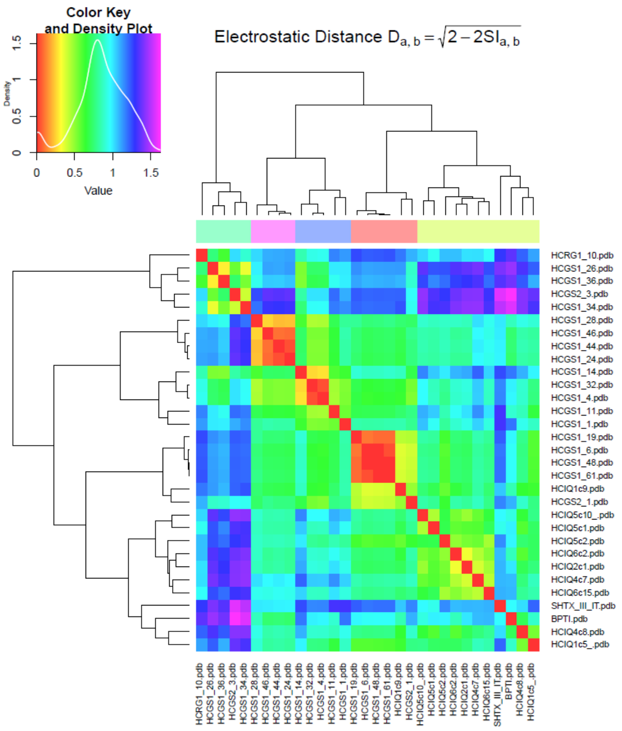

Figure S1. Electrostatic distances calculated from the similarity indices for the electrostatic potentials of HCIQ and HCGS peptides are shown in a color-coded matrix view (heat map). Red/orange colors indicate similar potentials, whereas blue color marks proteins with more distant electrostatic potentials. The tree along the side of the image group the proteins into groups of similar electrostatic potentials. Clustering of the peptides was conducted using webPIPSA 1.5 server (http://pipsa.h-its.org/).

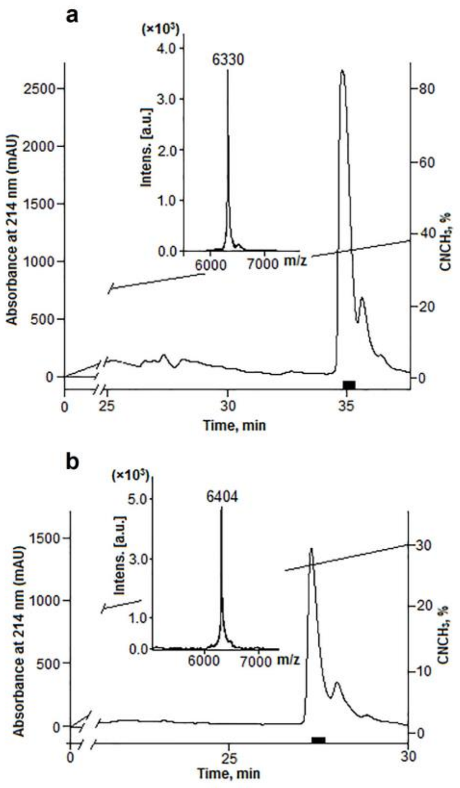

Figure S2. Purification of recombinant HCIQ peptides. HPLC elution profiles of rHCIQ2c1 (**A**) and rHCIQ4c7 (**B**) on reverse-phase column Jupiter C4 (250×10mm), using a linear gradient of ACN concentration (0–70% in 70 min) with 0.1% TFA and flow rate of 1.5 ml/min. Insert: MALDI-TOF/MS spectra and molecular masses of the peptides after RP-HPLC.

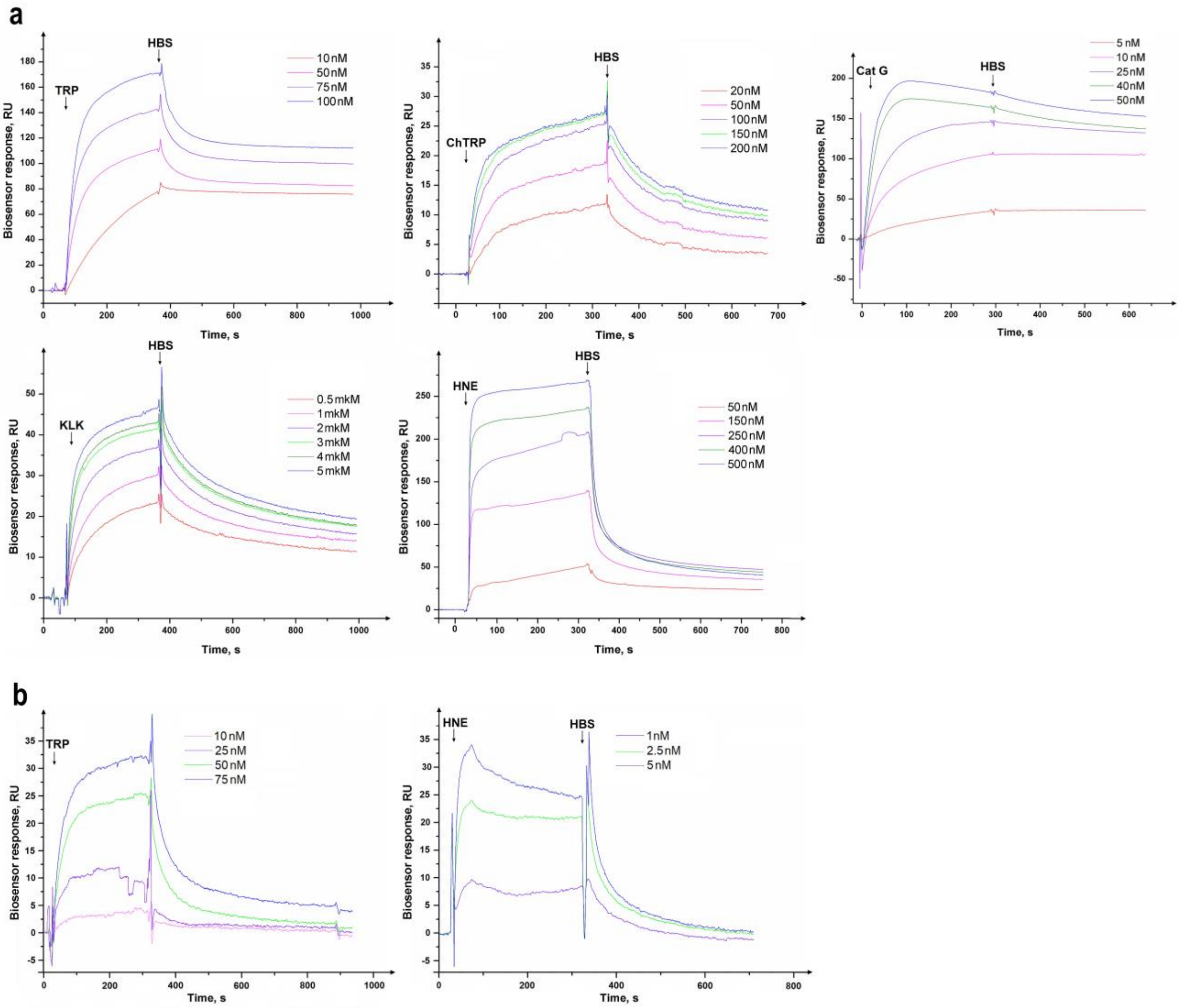

Figure S3. Binding sensograms of the immobilized rHCIQ2c1 (A) and rHCIQ4c1 (B) with serine proteinases at 25 °C. TRP – trypsin; ChTRP – α-chymotrypsin; Cat G – cathepsin G; KLK – kallikrein; HNE – human neutrophil elastase; HBS – HEPES buffered saline.
